# A case report of thalidomide in the treatment of camrelizumab-induced reactive cutaneous capillary hyperplasia

**DOI:** 10.1097/MD.0000000000034120

**Published:** 2023-06-30

**Authors:** Shujuan Fu, Cunya Li, Zhiying Wang, Zhixian Zhong, Yi Zhong

**Affiliations:** a Oncology Department, Shanghai TCM-Intergrated Hospital Shanghai University of Traditional Chinese Medicine, Shanghai, China; b Graduate School, Shanghai University of Traditional Chinese Medicine, Shanghai, China; c East Hospital Affiliated to Tongji University School of Medicine, Shanghai, China.

**Keywords:** camrelizumab, immune checkpoint inhibitors, reactive cutaneous capillary endothelial proliferation, thalidomide

## Abstract

Reactive cutaneous capillary endothelial proliferation (RCCEP) is the most prevalent immune-related adverse event observed in patients undergoing camrelizumab treatment, and there is a lack of effective therapeutic strategies. Thalidomide (THD) has anti-inflammatory, immunomodulatory, antiangiogenic, and antitumor properties, which have led to its use in various autoimmune diseases, hematological malignancies, solid tumors, and other disorders.

**Patient concerns:**

A 52-year-old male patient with lung cancer developed vascular moles on his face, neck, and back after 3 cycles of chemotherapy comprising pemetrexed and carboplatin combined with camrelizumab immunotherapy. The moles ranged in size (0.1–1.2 cm) and were red or red-black, appearing on the skin’s surface. The patient was advised to avoid scratching or friction, continue monitoring, and apply Yunnan Baiyao powder if a papule ruptured. After the third treatment cycle, papules on the patient’s face, particularly an eyelid vascular mole, ulcerated, causing significant psychological distress.

**Diagnosis:**

RCCEP induced by camrelizumab was considered.

**Interventions:**

The patient received 50 mg of THD in the morning and 100 mg in the evening.

**Outcomes:**

The vascular nevus began to shrivel after 1 week and started disappearing after 2 weeks of THD treatment. After 3 THD treatment courses, RCCEP was alleviated without recurrence, allowing the patient to successfully complete camrelizumab treatment.

**Lessons:**

During treatment with camrelizumab, if a patient develops moderate or severe RCCEP, and local therapy or anti-infective therapies prove insufficient, THD may be considered as a potential treatment option to improve RCCEP symptoms.

## 1. Introduction

Immune checkpoint inhibitors have ushered in a new era of cancer immunotherapy. However, the resulting immune-related adverse events (irAEs) present challenges for clinical applications. IrAEs are a significant concern when using immunotherapy alone or in combination with other treatments, including chemotherapy, targeted therapy, and additional immunotherapies, and may even lead to treatment-related fatalities. The most frequently reported irAE associated with camrelizumab is reactive cutaneous capillary endothelial proliferation (RCCEP), which has a high incidence and is dose-dependent.^[1–3]^ This case report presents evidence for thalidomide (THD) as a treatment strategy for RCCEP, offering a clinical direction for managing irAEs related to antitumor immunotherapy.

## 2. Case report

**Figure 1.**
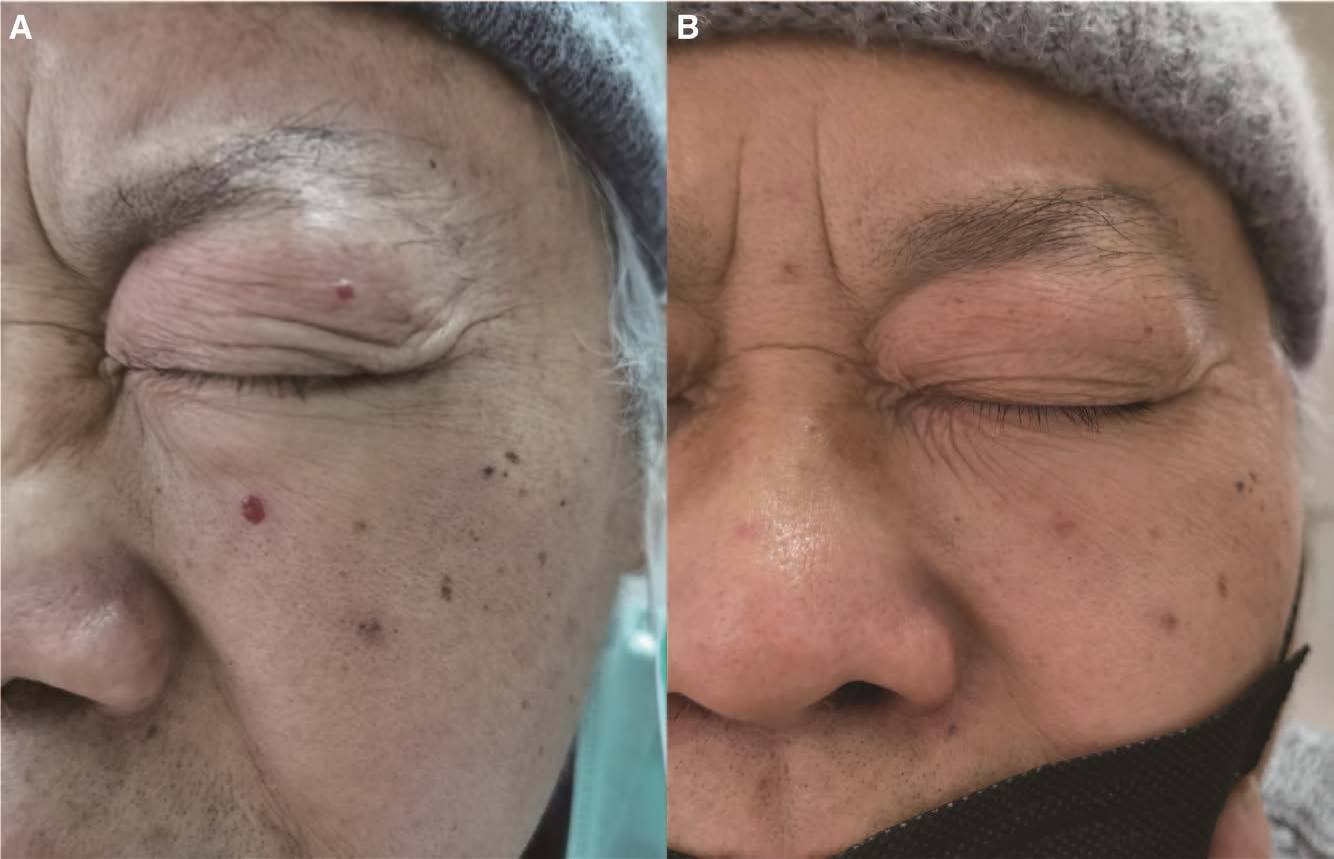
Reactive cutaneous capillary endothelial proliferation was relieved after thalidomide treatment. (A) Vascular nevus on the patient’s face before thalidomide treatment. (b) Vascular nevus shriveled after thalidomide treatment.

A 52-year-old male patient with lung cancer underwent tumor resection in August 2021 and began chemotherapy comprising pemetrexed and carboplatin combined with camrelizumab immunotherapy 1 month post-surgery. After 2 treatment cycles, scattered, dot-like, bright red circular papules emerged on the patient’s face, chest wall, and neck, with their numbers continually increasing. These were considered reactive capillary hyperplasia. Initially, no specific treatment was administered. The patient was advised to avoid scratching or friction, continue monitoring, and apply Yunnan Baiyao powder if a papule ruptured. After the third treatment cycle, the patient’s facial papules, particularly the eyelid vascular mole, ulcerated, causing significant psychological distress. The patient was subsequently given 50 mg of THD in the morning and 100 mg in the evening as further treatment. The patient reported that the vascular nevus appeared to shrivel after 1 week and began to disappear after 2 weeks (Fig. 1). After 3 THD treatment courses, RCCEP was alleviated without recurrence, allowing the patient to successfully complete camrelizumab treatment.

## 3. Discussion

Camrelizumab is a commonly used monoclonal antibody against programmed death protein 1. It is a Chinese-developed immune checkpoint inhibitor with established clinical efficacy. RCCEP is the most common irAE observed in camrelizumab treatment but has rarely been reported with other programmed death protein 1 inhibitors. RCCEP is often mislabeled as “capillary hemangioma”; however, the 2 conditions differ in terms of etiology, pathogenesis, morphology, pathological features, treatment efficacy, and clinical outcomes.

The exact pathogenesis of RCCEP remains unclear, but it is known to involve immune and angiogenesis mechanisms. Some studies have suggested that camrelizumab reactivates CD4+/CD8 + T lymphocytes, which attack skin antigens, causing inflammatory changes in skin tissue.^[2]^ Some scholars speculate. Other researchers propose that the reactivation of the immune response might result from the disruption of the balance between angiogenic factors and inhibitory vascular growth factors, leading to abnormal proliferation of skin capillary endothelial cells as an immune stress response.^[2]^ It is reported in the literature that there is a high incidence of RCCEP in patients treated with camrelizumab alone.^[4]^ The incidence of RCCEP decreased when camrelizumab was combined with chemotherapy and was lowest when camrelizumab was combined with anti-angiogenic drugs.^[5]^ Wang Feng^[3]^ found that the response rate to camrelizumab in patients with RCCEP was higher than that of patients without RCCEP, although there was no statistically significant difference. Some studies have also found that the objective effective rate of tumor treatment in patients with RCCEP after using camrelizumab is 28.9%, while patients without RCCEP exhibit no response to the drug.^[1]^

To address this issue, the Guidelines Working Committee of the Chinese Society of Clinical Oncology has developed and released the Expert Consensus on the Clinical Diagnosis and Treatment of Reactive Cutaneous Capillary Hyperplasia Induced by Camrelizumab.^[6]^ The consensus states that combining camrelizumab with antiangiogenic drugs or chemotherapeutic agents can significantly reduce the incidence of RCCEP. Low-dose antiangiogenic drugs, particularly apatinib, are among the therapeutic methods to improve RCCEP symptoms. THD, developed by the German pharmaceutical company Grantai and introduced to clinical use in 1957, was withdrawn from the pharmaceutical market in 1961 due to its teratogenic and neurotoxic side effects. However, following >2 decades of research, THD has been widely employed in various autoimmune diseases, hematological malignancies, solid tumors, and other conditions. THD exhibits anti-inflammatory,^[7]^ immune-regulatory,^[8]^ angiogenesis-inhibitory, and antitumor properties.^[9,10]^

Price et al^[11]^ identified the in vivo metabolite 5’-OH-thalidomide of THD and confirmed the latter’s antiangiogenic activity in a murine arterial model. Shen Mei et al^[12]^ reported that THD could significantly inhibit the growth and angiogenesis of human ovarian cell carcinoma subcutaneously transplanted tumors in nude mice. The tumor volumes in the experimental group and control group were 649.72 ± 274.39 and 1086.15 ± 262.25 mm³, respectively (*P* < .01). Additionally, ongoing studies are investigating THD derivatives, such as lenalidomide. THD inhibits vascular endothelial growth factor and fibroblast growth factor, reducing endothelial cell proliferation and suppressing angiogenesis. Tumor growth and metastasis are closely related to angiogenesis, so THD indirectly produces antitumor effects by inhibiting angiogenesis.

In this case, the patient declined to use antiangiogenic drugs for financial reasons, leading to the proposal of employing THD, an older medication, in a new capacity. After obtaining the patient’s informed consent, we administered 50 mg of THD in the morning and 100 mg in the evening. The patient reported that the vascular nevus appeared shriveled after 1 week and began to disappear after 2 weeks.

**Figure 2.**
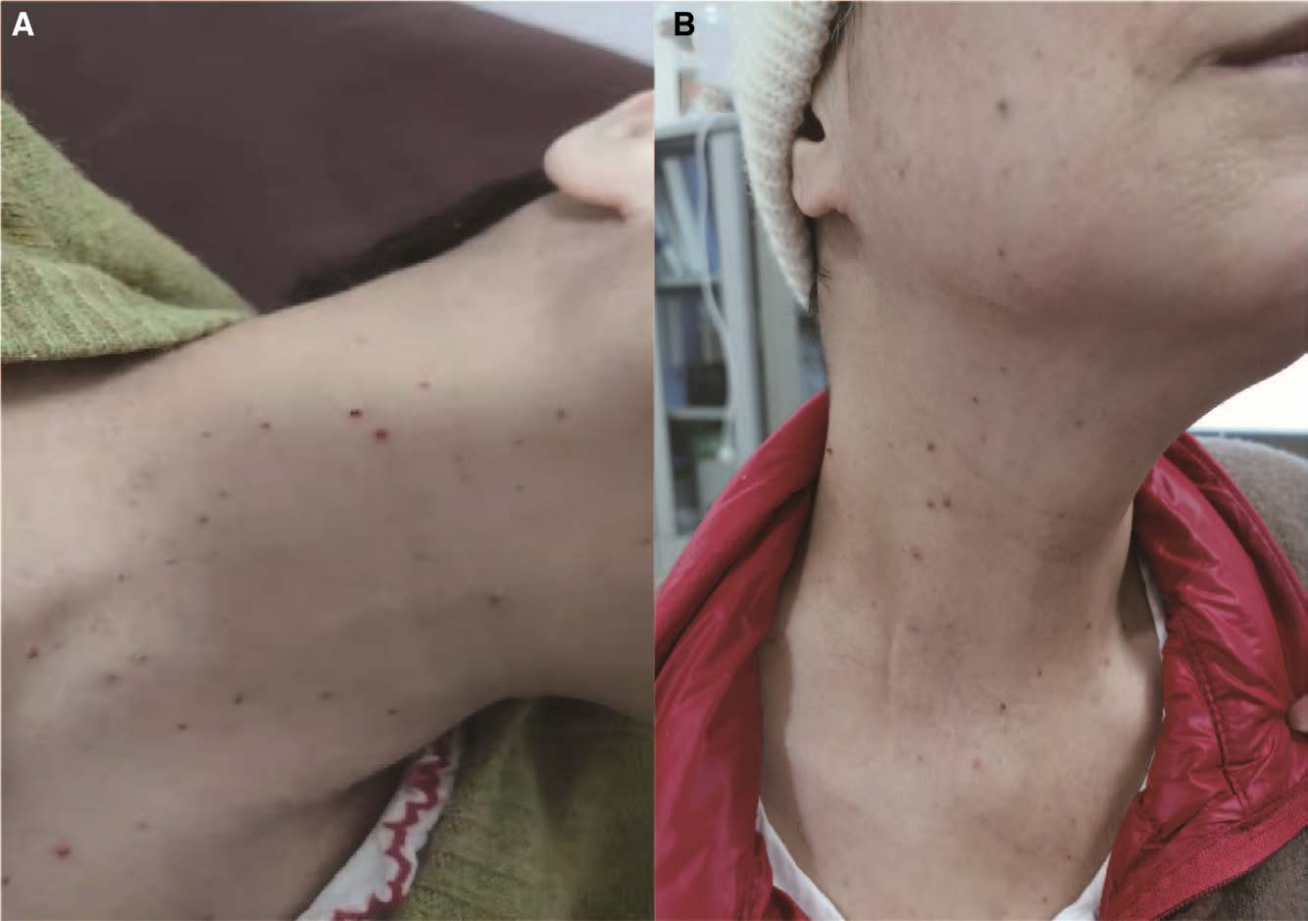
Another case of reactive cutaneous capillary endothelial proliferation relieved after thalidomide treatment. (A) Vascular nevus on the patient’s neck before thalidomide treatment. (B) Vascular nevus shriveled after thalidomide treatment.

Upon reviewing clinical reports on the combined use of THD to treat RCCEP and the successful treatment of this case, we continued to apply the treatment to several other patients and observed similar vascular nevus regression and symptom improvement (Fig. 2).

## 4. Conclusion

In conclusion, the safe use of THD, with proper risk control, is beneficial, as it not only achieves “attenuation and synergy,” alleviating RCCEP but also enhances antitumor activity. During camrelizumab treatment, if a patient develops moderate or severe RCCEP and local therapy or anti-infective treatment is inadequate, adding THD may be considered as a potential treatment method to improve RCCEP symptoms. Further experimental and clinical research will be conducted to provide additional insight into this approach.

## Author contributions

**Data curation:** Shujuan Fu, Cunya Li, Yi Zhong.

**Formal analysis:** Cunya Li.

**Investigation:** Zhiying Wang, Zhixian Zhong.

**Writing – original draft:** Shujuan Fu, Yi Zhong.
